# Cortical and subcortical structural differences in psychostimulant-free ADHD youth with and without a family history of bipolar I disorder: a cross-sectional morphometric comparison

**DOI:** 10.1038/s41398-023-02667-0

**Published:** 2023-11-30

**Authors:** Ziyu Zhu, Du Lei, Kun Qin, Maxwell J. Tallman, L. Rodrigo Patino, David E. Fleck, Qiyong Gong, John A. Sweeney, Melissa P. DelBello, Robert K. McNamara

**Affiliations:** 1 Huaxi MR Research Center (HMRRC), Department of Radiology, West China Hospital of Sichuan University, Chengdu, 610041 PR China; 2 Department of Psychiatry and Behavioral Neuroscience, University of Cincinnati College of Medicine, Cincinnati, 45219 OH USA; 3 College of Medical Informatics, Chongqing Medical University, Chongqing, 400016 PR China; 4 Research Unit of Psychoradiology, Chinese Academy of Medical Sciences, Chengdu, Sichuan PR China; 5 Department of Radiology, Taihe Hospital, Hubei University of Medicine, Shiyan, 442012 PR China; 6 Department of Radiology, West China Xiamen Hospital of Sichuan University, Xiamen, 361021 Fujian PR China

**Keywords:** ADHD, Prognostic markers

## Abstract

Although attention-deficit/hyperactivity disorder (ADHD) and a family history of bipolar I disorder (BD) are associated with increased risk for developing BD, their neuroanatomical substrates remain poorly understood. This study compared cortical and subcortical gray matter morphology in psychostimulant-free ADHD youth with and without a first-degree relative with BD and typically developing healthy controls. ADHD youth (ages 10-18 years) with (‘high-risk’, HR) or without (‘low-risk’, LR) a first-degree relative with BD and healthy comparison youth (HC) were enrolled. High-resolution 3D T1-weighted images were acquired using a Philips 3.0 T MR scanner. The FreeSurfer image analysis suite was used to measure cortical thickness, surface area, and subcortical volumes. A general linear model evaluated group differences in MRI features with age and sex as covariates, and exploratory correlational analyses evaluated associations with symptom ratings. A total of *n* = 142 youth (mean age: 14.16 ± 2.54 years, 35.9% female) were included in the analysis (HC, *n* = 48; LR, *n* = 49; HR, *n* = 45). The HR group exhibited a more severe symptom profile, including higher mania and dysregulation scores, compared to the LR group. For subcortical volumes, the HR group exhibited smaller bilateral thalamic, hippocampal, and left caudate nucleus volumes compared to both LR and HC, and smaller right caudate nucleus compared with LR. No differences were found between LR and HC groups. For cortical surface area, the HR group exhibited lower parietal and temporal surface area compared with HC and LR, and lower orbitofrontal and superior frontal surface area compared to LR. The HR group exhibited lower left anterior cingulate surface area compared with HC. LR participants exhibited greater right pars opercularis surface area compared with the HC. Some cortical alterations correlated with symptom severity ratings. These findings suggest that ADHD in youth with a BD family history is associated with a more a severe symptom profile and a neuroanatomical phenotype that distinguishes it from ADHD without a BD family history.

## Introduction

The onset of bipolar I disorder (BD) often occurs during late childhood and adolescence [1], and is commonly preceded by attention-deficit/hyperactivity disorder (ADHD) [2, 3]. ADHD prevalence rates in youth with BD are substantially higher than the general population, particularly in pre-pubescent children [4–6], and antecedent ADHD increases the risk for developing BD [7]. Additionally, BD and ADHD share overlapping genetic liability [8, 9], and having a first-degree relative with BD is a robust risk factor for both BD [10–12] and ADHD [13, 14]. Youth with ADHD and a first-degree relative with BD also present with more severe symptom profiles, including more severe ADHD hyperactivity/impulsivity, mania, and depression, as well as parent-reported ratings of emotional dysregulation compared with ADHD youth with healthy parents [15, 16]. These findings suggest that ADHD in conjunction with BD familial risk may represent a different phenotype that confers greater risk for developing BD in a subset of individuals. However, the underlying regional neuroanatomical substrates associated with elevated BD risk in youth with ADHD and BD familial risk remain poorly understood.

The initial onset of both ADHD and BD commonly occurs during a developmental period associated with progressive cortical and subcortical structural maturational changes [17–19]. Consistent with a perturbation in maturational trajectories, meta-analyses of cross-sectional structural imaging studies indicate that youth with ADHD or first-episode BD exhibit regional alterations in cortical and subcortical gray matter volumes compared with typically developing youth. Specifically, ADHD youth exhibit decreased gray matter volumes in bilateral frontal gyri, bilateral superior temporal gyri, amygdala and hippocampus, and increased gray matter volumes in left middle occipital gyrus and striatum [20, 21], while BD youth exhibit decreased gray matter volumes in anterior cingulate cortex (ACC), medial superior frontal gyrus and gyrus rectus, and increased gray matter volumes in posterior cingulate cortex (PCC) and striatum [22, 23]. Neuroimaging studies have also provided evidence for delayed development of cortical thickness and surface area in youth with ADHD [24, 25] and first-episode BD [26], particularly in frontal-temporal regions, and associated alterations in network connectivity [27–29] involved in emotional regulation and cognition [30].

Previous studies have linked genetic risk for BD to variations in brain structure [31, 32], and that unaffected subjects with a BD family history exhibit region-specific structural alterations compared with both healthy controls and first-degree relatives with BD [23, 33–35]. However, the latter studies did not control for ADHD comorbidity or psychostimulant exposure which has been shown to normalize regional structural deficits in ADHD youth [36–39]. Although studies have found that regional volumetric and cortical thickness alterations unique to ADHD or BD are both exhibited in adults with co-occurring BD and ADHD [40, 41], to our knowledge no studies have directly compared structural metrics in unaffected psychostimulant-free ADHD youth with and without a first-degree BD relative. To address this, the present study investigated regional cortical and subcortical gray matter morphology in psychostimulant-free ADHD youth with and without a first-degree relative with BD, and healthy comparison youth. Based on previous neuroanatomical evidence, we hypothesized that ADHD youth with and without a BD family history would exhibit overlapping cortical and subcortical structural abnormalities compared with healthy controls, and that ADHD youth with a BD family history would additionally exhibit unique structural abnormalities compared to ADHD youth without a BD family history. Exploratory analyzes investigated associations between morphological measures and symptom severity ratings.

## Methods

### Participants

Three groups of psychostimulant-free youth (10-18 years) were recruited: 1) youth with ADHD and at least one biological parent or sibling with BD (‘high-risk’, HR), 2) youth with ADHD and no first- or second-degree relative with a mood or psychotic disorder (‘low-risk’, LR), and 3) typically developing healthy comparison youth (HC) with no personal or family history of a DSM-5 Axis I psychiatric disorder. Family Interview for Genetics Studies (FIGS) [42] was used to identify suspected BD diagnoses in first- or second-degree relatives including siblings and to determine the absence of mood or psychotic disorders in the comparison groups. For the HR group, the Structured Clinical Interview for DSM-5 (SCID-5-CV) confirmed a parental diagnosis of BD [43]. Pubertal status was determined with the Duke Tanner Stage Self-assessment [44], and handedness determined with the Crovitz Handedness Questionnaire [45]. Subjects were excluded if they had any contraindication to an MRI scan (e.g., braces or claustrophobia), had an IQ < 80 as determined by the Wechsler Abbreviated Scale of Intelligence (WASI) [46], had a current or past major medical or neurological illness that could influence MRI results; had a history of head trauma with loss of consciousness (>10 min); had a lifetime DSM-5 substance use disorder. All subjects were assessed using the Kiddie Schedule for Affective Disorders and Schizophrenia (KSADS-PL) [47] to determine the presence of ADHD (all subtypes) and to confirm the absence of DSM-5 mood, conduct, eating, psychotic disorders, Tourette’s disorder, chronic tic disorder, or autism spectrum disorder. Subjects were required to have had no exposure to psychostimulants (prescription or recreational) or other medications used for the treatment of ADHD (e.g. atomoxetine) for at least 3 months prior to screening, had no lifetime exposure to mood-stabilizer or antipsychotic medications, and had no clinically significant ECG or blood pressure abnormalities.

All participants and their legal guardians provided written informed consent/assent. The research protocol was approved by the Institutional Review Board of the University of Cincinnati, and the study was registered on clinicaltrials.gov (NCT02478788).

### Symptom ratings

ADHD symptom ratings were obtained using the clinician-administered Attention-Deficit Hyperactivity Disorder Rating Scale (ADHD-RS) [48], and inattention and hyperactivity/impulsivity subscale scores were analyzed separately. Depression symptom severity was determined using the Children’s Depression Rating Scale-Revised (CDRS-R) [49, 50], and manic symptom severity was determined using the Young Mania Rating Scale (YMRS) [51]. Global functioning was assessed using the Children’s Global Assessment Scale (CGAS) [52]. ADHD youth were also rated using the Clinical Global Impression-Severity Scale (CGI-S) to assess overall illness severity [53]. All clinician ratings were administered by blinded child and adolescent psychiatrists or psychologists with established inter-rater reliabilities (kappa>0.9). Parents completed the Child Behavior Checklist (CBCL ages 6-18 2001) [54], and CBCL total score, internalization and externalization subscale scores, and Dysregulation Profile (CBCL-DP) scores (i.e., the sum of the attention, aggression, and anxious/depressed scores) were assessed.

### MRI acquisition

High-resolution 3D T1-weighted images were collected using a Philips Ingenia 3 T MRI scanner with a 32-channel head coil. Sequence parameters were as follows: TR = 8.1 ms, TE = 3.7 ms, flip angle = 8°, field of view = 256 × 224, matrix = 256 × 224, voxel size = 1 × 1 × 1mm^3^, number of axial slices = 160, gap between slices = 0. After the MR scan, data with excessive head movements, brain lesions or obvious artifacts were discarded. Images were inspected by neuroradiologists who made decisions about excessive motion artifact for scan inclusion.

### Image processing

All structural MRI scans were processed on the same workstation using FreeSurfer version 6.0.0 (http://surfer.nmr.mgh.harvard.edu/) to obtain unbiased estimates of morphometric measures, including surface area, cortical thickness, and subcortical volumes. Briefly, the procedure includes intensity normalization; removal of non-brain tissue; segmentation of cortical grey, subcortical white and deep grey matter structures; and triangular tessellation of the grey/white matter interface and white matter/cerebrospinal fluid (CSF) boundary (pial surface). All surface reconstructions were visually inspected and, where necessary, corrected manually using editing tools provided by FreeSurfer, including corrections of erroneous skull stripping and white matter and grey matter segmentations. Individual reconstructed surfaces were smoothed, transformed and resampled onto a common standard space. Regions based on the Desikan-Killiany atlas [55] were segmented, which resulted in cortical thickness and surface area values for 34 left and 34 right hemisphere regions, as well as the volume of 14 bilateral subcortical regions (i.e. thalamus, amygdala, caudate, putamen, pallidum, hippocampus and accumbens).

### Statistical analyses

Tests for group differences in demographic and descriptive variables were performed using SPSS (IBM SPSS Statistics V23.0). We conducted one-way analysis of variance (ANOVA) and chi-square tests to compare continuous and categorical variables across groups. Independent-sample t-tests and chi-square tests were performed for pairwise comparisons. Statistical analyses of the morphometric measures were performed using *R* software (Version 4.1.2, http://www.r-project.com). Extracted values for cortical thickness, surface area and subcortical volumes of all participants were fitted into three independent analysis of covariance (ANCOVA) models as independent variables, with group (HR, LR, HC) as a fixed factor. As age and gender are known to impact brain morphometry, they were used as covariates. All group differences were deemed significant with a threshold level of the false discovery rate (FDR) corrected *p* < 0.05. Post-hoc two-sample comparisons were performed if ANCOVA was significant. For all morphological measures that differed statistically between the LR and/or HR groups and HC, we fit a regression model including each morphological measures as outcome, and each clinical rating, group and their interaction term (group × clinical score) as predictors. Whether the clinical association between two ADHD groups differed significantly was also determined by the significance level of regression coefficients of the interaction term (*p* < 0.01 uncorrected). Additionally, we performed partial correlation analyses (correcting for age and sex) to examine correlations between morphological measures and clinical scores. A two-sided *p* value < 0.05 was used as the criterion to indicate a statistically significant difference.

## Results

### Demographic and clinical characteristics

A total of *n* = 142 adolescents (mean age: 14.16 ± 2.54 years, 35.9% female) were included in the analysis (HC, *n* = 48; LR, *n* = 49; HR, *n* = 45). Sociodemographic and clinical characteristics are presented in Table 1. No statistically significant group differences were observed for age, sex, or handedness, or for prior psychostimulant exposure in the ADHD groups. Both HR and LR ADHD groups differed significantly from HCs on all ratings (all *p*s < 0.0001). Compared with the LR group, the HR group had higher ADHD-RS hyperactivity/impulsivity subscale scores (*p* = 0.032), YMRS (*p* = 0.004), and CGI-S (*p* = 0.014) total scores, and higher CBCL total score (*p* = 0.002) and internalization (*p* = 0.010), externalization (*p* = 0.002), and dysregulation (*p* = 0.047) subscale scores.

**Table 1:** Demographic and Clinical Characteristics.

Variable^a^	Healthy Controls	Low-Risk	High-Risk	Omnibus statistic, *P* value^b^	Low-risk vs. High-risk
	(*n* = 48)	(*n* = 49)	(*n* = 45)		*P* value^c^
Age, years	14.6 ± 2.45	14.0 ± 2.55	13.8 ± 2.62	0.362	0.690
Gender, *n* (%) male	29 (60.4)	33 (67.3)	29 (64.4)	0.775	0.767
Handedness, *n* (%) right	46 (95.8)	40 (81.6)	40 (88.9)	0.087	0.324
CDRS-R Total Score	18.0 ± 2.3	24.0 ± 5.9	26.6 ± 7.7	**<0.001**	0.076
YMRS Total Score	0.75 ± 1.9	2.9 ± 3.3	5.3 ± 4.3	**<0.001**	**0.004**
CGAS Total Score	88.3 ± 5.9	52.6 ± 6.9	51.0 ± 7.2	**<0.001**	0.284
CGI-S	–	4.3 ± 0.6	3.9 ± 0.5	–	**0.014**
CBCL Total Score	7.7 ± 8.2	37.3 ± 17.9	53.2 ± 28.9	**<0.001**	**0.002**
CBCL Internalizing Subscale	2.4 ± 2.5	8.2 ± 6.3	12.5 ± 8.9	**<0.001**	**0.010**
CBCL Externalizing Subscale	1.7 ± 2.2	8.3 ± 6.8	14.9 ± 12.2	**<0.001**	**0.002**
CBCL Dysregulation Subscale	3.4 ± 3.9	20.9 ± 9.6	25.9 ± 13.5	**<0.001**	**0.047**
ADHD-R Total Score	3.2 ± 3.9	33.5 ± 10.1	35.8 ± 10.8	**<0.001**	0.281
Inattention Subscale	1.8 ± 2.3	21.0 ± 4.8	19.9 ± 5.8	**<0.001**	0.299
Hyperactivity/Impulsivity Subscale	1.3 ± 2.1	12.5 ± 8.1	15.9 ± 7.3	**<0.001**	**0.032**
ADHD type					
ADHD-I, *n* (%)	–	28 (57.1)	11 (24.4)		**0.001**
ADHD-H, *n* (%)	–	–	1 (2.2)	–	\–
ADHD-C, *n* (%)	–	21 (42.9)	33 (73.3)		**0.003**
Prior psychostimulant exposure, *n* (%)	–	16 (32.6)	18 (40.0)	–	0.459

^a^Values are group mean ± S.D. or number of subjects (*n*) and percent (%).

^b^One-way ANOVA or *X*^2^.

^c^t-test or *X*^2^.

Bold values indicates statistically significant *p* values less than 0.05.

### Neuroanatomical differences

**Fig. 1:**
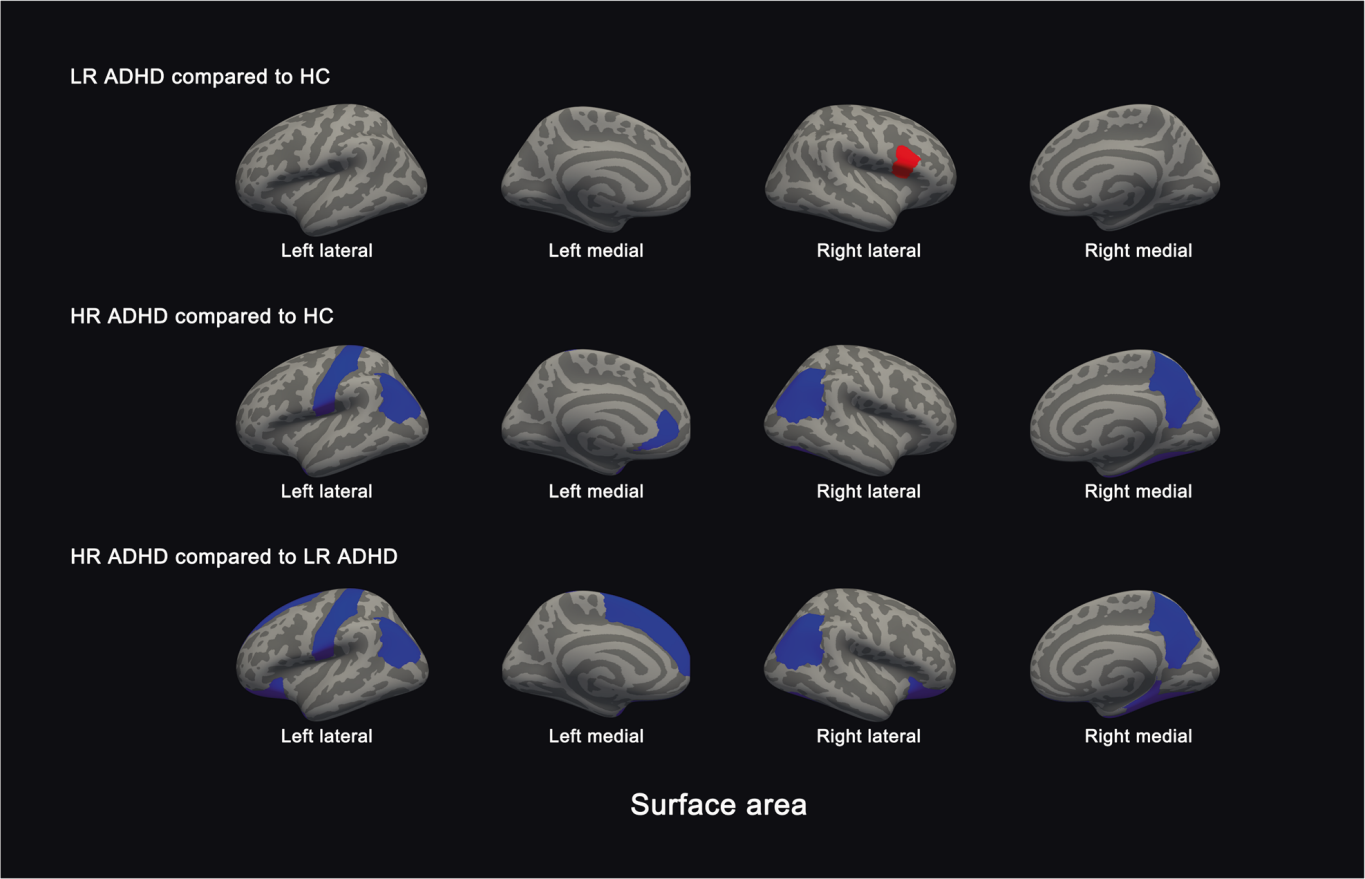
Cortical regions exhibiting differences in surface area in high-risk ADHD youth (*n* = 45), low-risk ADHD youth (*n* = 49), and healthy controls (HC, *n* = 48) (*p* < 0.05, FDR-corrected). Colored regions show significant differences in surface area between the 2 groups with red representing increase and blue representing decrease.

**Fig. 2:**
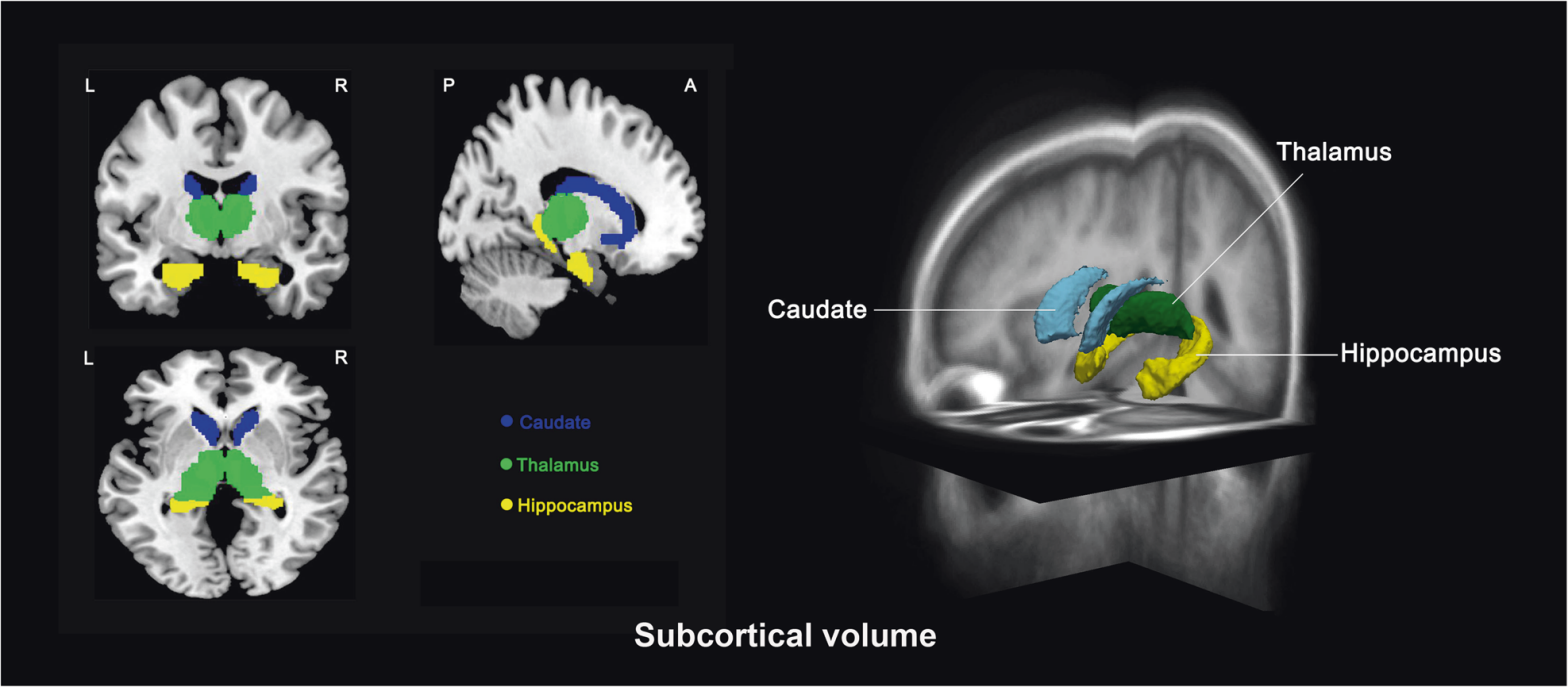
Subcortical regions exhibiting volumetric differences in high-risk ADHD youth (*n* = 45), low-risk ADHD youth (*n* = 49), and healthy controls (HC, *n* = 48). Significant differences in the volumes of the bilateral thalamus, caudate, and hippocampus were found among the three groups (*p* < 0.05, FDR-corrected).

For cortical surface area, the HR group exhibited smaller left postcentral, bilateral inferior parietal, right precuneus extending to the right PCC, right left temporal pole, right fusiform, and left ACC surface area compared with the HC group, and smaller bilateral lateral orbitofrontal, left superior frontal, left postcentral, bilateral inferior parietal, right precuneus extending to the right PCC, left temporal pole, right fusiform, and right parahippocampal cortical surface area compared with the LR group (Fig. 1, Table 2, *p* < 0.05, FDR corrected). The LR group exhibited a larger right pars opercularis compared with the HC group (Table 2, *p* = 0.001, FDR corrected). Group differences in cortical thickness did not survive correction for multiple comparisons. For subcortical volumes, the HR group exhibited smaller bilateral thalamus, caudate nuclei, and hippocampal volumes compared with the LR group as well as the HC group with the exception of the right caudate, and there were no significant differences between LR and HC groups (Fig. 2, Table 2, *p* < 0.05, FDR corrected). In an exploratory follow-up analysis, we examined associations among regional measurements with significant differences between the LR and HR groups. This involved computing the correlation matrices of these relations separately for volume and surface area measurements. These matrix plots are presented for heuristic purposes in the Supplementary materials. Overall, associations were almost exclusively positive. We compared these associations between groups, and found no significant differences between the LR and HR groups after correction for multiple comparisons.

**Table 2:** Group differences in cortical surface area and subcortical volumes.

Region^a^	ANCOVA	*P*-value^b^		
		Low-Risk > HC	High-Risk < HC	High-Risk < Low-Risk
**Subcortical volume**				
Left thalamus	0.001	0.855	**0.001**	**0.021**
Right thalamus	0.008	1.000	**0.016**	**0.024**
Left caudate	0.006	1.000	**0.015**	**0.015**
Right caudate	0.016	1.000	0.104	**0.017**
Left hippocampus	0.013	1.000	**0.035**	**0.028**
Right hippocampus	0.012	1.000	**0.019**	**0.048**
**Surface area**				
Left lateral orbitofrontal cortex	0.005	0.620	0.128	**0.003**
Right lateral orbitofrontal cortex	0.008	0.954	0.116	**0.007**
Left superior frontal cortex	0.004	1.000	0.052	**0.004**
Right pars opercularis cortex	0.001	**0.001**	0.320	0.119
Left postcentral cortex	0.004	1.000	**0.022**	**0.005**
Left inferior parietal cortex	0.005	1.000	**0.023**	**0.008**
Right inferior parietal cortex	0.005	1.000	**0.023**	**0.008**
Right precuneus cortex extending to right PCC	0.003	1.000	**0.022**	**0.004**
Left temporal pole cortex	0.003	1.000	**0.006**	**0.012**
Right fusiform cortex	0.001	1.000	**0.005**	**0.002**
Right parahippocampal cortex	0.002	1.076	0.639	**0.002**
Left anterior cingulate cortex	0.004	0.359	**0.003**	0.190

^a^Regions exhibiting significant group differences (*p* < 0.05, FDR corrected).

^b^Post hoc pairwise comparison: *p* < 0.05, FDR-corrected (bolded).

### Associations with symptom ratings

**Fig. 3:**
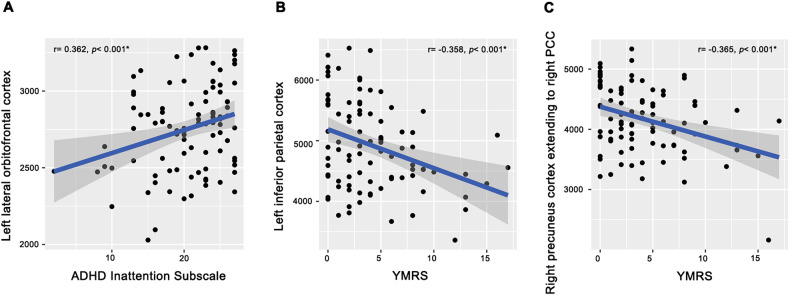
Linear correlations between affected regions and symptom severity scores among low-risk and high-risk ADHD youth (*n* = 94). ADHD inattention subscale scores were positively correlated with left lateral orbitofrontal surface area (**A**), and YMRS total score was negatively correlated with surface area of the left inferior-parietal (**B**) and right precuneus extending to the right PCC (**C**).

**Fig. 4:**
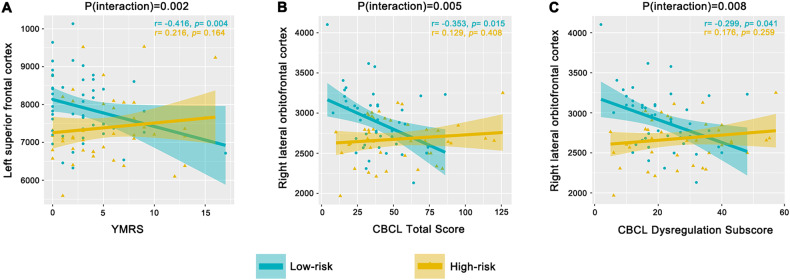
Interactions (group × clinical score) between low-risk ADHD youth (*n* = 49) and high-risk ADHD youth (*n* = 45). Within ADHD groups, nominally significant group interactions were observed for relationships between YMRS totalscores and left superior frontal surface area (*p* = 0.002, uncorrected) (**A**), CBCL total scores and right lateral orbitofrontal surface area (*p* = 0.005, uncorrected) (**B**), and CBCL dysregulation subscale scores and right lateral orbitofrontal surface area (*p* = 0.008, uncorrected) (**C**).

Among both LR and HR groups (*n* = 94), YMRS total scores correlated negatively with the surface area both left inferior parietal (*r* = −0.358, *p* < 0.001, FDR corrected) and right precuneus extending to the right PCC (*r* = −0.365, *p* < 0.001, FDR corrected), and ADHD-RS inattention subscale scores correlated positively with left lateral orbitofrontal surface area (*r* = 0.362, *p* < 0.001, FDR corrected) (Fig. 3). Nominally significant interactions between LR and HR groups were observed for correlations between YMRS total scores and left superior frontal surface area (*p* = 0.002, uncorrected) (Fig. 4A), CBCL total score and right lateral orbitofrontal surface area (*p* = 0.005, uncorrected) (Fig. 4B), and CBCL dysregulation subscale scores and right lateral orbitofrontal surface area (p = 0.008, uncorrected) (Fig. 4C). In each case, these were inversely correlated in the LR group but not in the HR group. No significant correlations with symptom ratings or group interactions were found in other regions exhibiting significant group differences.

## Discussion

The primary aim of this study was to compare neuroanatomical features in psychostimulant-free ADHD youth with (high-risk) and without (low-risk) a BD family history. In agreement with our hypothesis, we found that HR youth exhibited regional subcortical and cortical deficits compared with LR youth as well as healthy youth. Furthermore, the LR group did not exhibit any significant regional subcortical and cortical deficits compared with HC. The HR group also exhibited a more severe symptom profile, including higher ADHD hyperactivity/impulsivity subscale scores, mania scores, and dysregulation subscale scores, compared with the LR group. Among both LR and HR groups, regional cortical morphological differences correlated with some symptom ratings including mania and dysregulation. Taken collectively, these findings suggest that ADHD in conjunction with BD family history is associated with a more a severe symptom profile and a more pervasive pattern of morphological deficits than are ADHD participants without a BD family history.

In contrast to prior meta-analyses [20, 21], low-risk ADHD subjects did not exhibit any significant cortical thickness or subcortical deficits compared with HC. The only significant morphological abnormality exhibited by the LR group relative to HC was their greater right pars opercularis surface area. The pars opercularis is a part of inferior frontal gyrus and plays a crucial role in inhibiting behavioral responses [56]. Although the reason(s) for this discrepancy is unclear, the majority of prior studies included ADHD youth that were being treated with psychostimulant medications, whereas a majority of ADHD youth in the present study were psychostimulant-naïve or had no exposure to psychostimulants for at least 3 months prior to scanning. However, prior imaging studies suggest that psychostimulant treatment attenuates regional gray matter deficits in ADHD subjects [36–39]. A second possibility is that previous ADHD studies did not control for BD family history, and the present and prior results [23, 33–35] indicate that BD family history is associated with robust regional structural abnormalities compared with healthy subjects.

Consistent with our hypothesis, our findings demonstrate that ADHD youth with, but not without, a BD family history exhibit widespread cortical and subcortical gray matter deficits compared with healthy comparison youth. Specifically, ADHD youth with a BD family history exhibited smaller bilateral thalamic, hippocampal, and left caudate volumes, and lower parietal, temporal, and ACC surface area compared with HC. Some of these regional deficits have previously been reported in youth with ADHD, including smaller caudate and hippocampal volumes [20, 21], as well as first-episode BD patients, including smaller temporal, parietal, orbitofrontal, and superior frontal cortical volumes [22, 23]. Moreover, unaffected individuals with a BD family history exhibited reduced orbitofrontal and superior frontal cortical volumes [33, 57]. It is notable that amygdala volumes, which are smaller in youth with BD [58] and ADHD [20], were not smaller in ADHD youth with or without a BD family history. However, this finding is consistent with the majority of previous studies in unaffected youth with a BD family history which did not control for ADHD comorbidity [33]. Collectively, these findings demonstrate that ADHD in conjunction with BD family history is associated with a more pervasive pattern of morphological deficits that are distinctive from those been in youth with ADHD without a BD family history.

Compared with ADHD youth without a BD family history, ADHD youth with a BD family history exhibited robust cortical and subcortical gray matter deficits in regions that contribute to the default mode network (DMN) [30, 59], including the hippocampus, lateral orbitofrontal cortex, superior frontal cortex, inferior parietal, temporal pole cortex, and right parahippocampal cortex. These results are in agreement with recent structural and functional neuroimaging studies, which have observed DMN abnormalities in both ADHD and BD [27–29]. Moreover, the DMN has increasingly been implicated in the pathophysiology of ADHD and BD due to its key role in self-referential processing and emotional regulation [30], and DMN dysregulation may contribute to elevated risk for developing BD in youth with a family history of BD [60–63]. Functional imaging studies are therefore warranted to interrogate DMN integrity in ADHD with and without familial risk for BD.

Compared with the LR group, the HR group also exhibited a more severe symptom profile, including higher ADHD hyperactivity/impulsivity subscale scores, mania scores and parent-rated externalization and dysregulation scores. Among both LR and HR groups, mania scores correlated negatively with surface area of both left inferior parietal and right precuneus extending to the right PCC, and ADHD inattention subscale scores correlated positively with left lateral orbitofrontal surface area. No significant correlations were observed between subcortical volumes and symptom ratings after controlling for age and sex. Within the LR group, negative correlations were observed between mania total scores and left superior frontal surface area, and CBCL total scores and dysregulation subscale scores with right lateral orbitofrontal surface area. In contrast, these correlations were not significant in HR. These findings suggest that regions that exhibit morphological differences in LR and HR ADHD groups are associated with symptom measures that are relevant to BD risk progression, including mania and dysregulation [2, 64].

The present study has several notable limitations. First, the sample size may be too small to detect more subtle structural differences after controlling for multiple comparisons. Second, the study was cross-sectional and prospective longitudinal studies are required to determine the relevance of these findings to BD risk progression. Third, this structural study does not inform on potential associations with cortical and subcortical function measures. Strengths of this study include a well-characterized cohort of psychostimulant-free ADHD youth with and without BD family history with similar group demographics, a healthy comparison group, stringent correction for multiple comparisons, and assessment of both cortical and subcortical morphology.

## Conclusion

The present cross-sectional findings demonstrate for the first time that psychostimulant-free ADHD youth with familial risk for BD exhibit robust regional cortical and subcortical morphological deficits compared with ADHD youth without familial risk for BD and healthy comparison youth. No significant reduction in cortical surface area or subcortical volumes was seen in the LR group. Gray matter deficits are consistent with a more pervasive disruption of peripubertal neurodevelopmental trajectories, and contributing heritable and illness-related biological features warrant additional investigation. Associations between regional morphological measures and symptom ratings including mania and dysregulation suggest potential relevance to BD risk progression in youth with ADHD and familial risk for BD.

### Supplementary information

Cortical and Subcortical Structural Differences in Psychostimulant-Free ADHD Youth With and Without a Family History of Bipolar I Disorder: A Cross-Sectional Morphometric Comparison

## Data Availability

The datasets used and/or analyzed during the current study are available from the corresponding author on reasonable request. The data are not publicly available due to privacy or ethical restrictions.
