# Supplementary material for: Cortical and subcortical structural differences in psychostimulant-free ADHD youth with and without a family history of bipolar I disorder: a cross-sectional morphometric comparison
Source: Transl Psychiatry. 2023 Nov 30;13:368. doi: 10.1038/s41398-023-02667-0 (PMC10689449; doi:10.1038/s41398-023-02667-0)
Supplement: Supplementary file 1 — Cortical and Subcortical Structural Differences in Psychostimulant-Free ADHD Youth With and Without a Family History of Bipolar I Disorder: A Cross-Sectional Morphometric Comparison [file 41398_2023_2667_MOESM1_ESM.docx]

**Supplementary material**

**Cortical and Subcortical Structural Differences in Psychostimulant-Free ADHD Youth With and Without a Family History of Bipolar I Disorder: A Cross-Sectional Morphometric Comparison**

Ziyu Zhu^1, 2#^, Du Lei^3#*^, Kun Qin^1, 2, 4, 5^, Maxwell J. Tallman^2^, L. Rodrigo Patino^2^, David E. Fleck^2^, Qiyong Gong^1,6*^, John A. Sweeney^1,2^, Melissa P. DelBello^2^, and Robert K. McNamara^2^

^1^Huaxi MR Research Center (HMRRC), Department of Radiology, West China Hospital of Sichuan University, Chengdu 610041, PR China

^2^Department of Psychiatry and Behavioral Neuroscience, University of Cincinnati College of Medicine, Cincinnati 45219, OH, USA

^3^College of Medical Informatics, Chongqing Medical University, Chongqing 400016, PR China

^4^Research Unit of Psychoradiology, Chinese Academy of Medical Sciences, Chengdu, Sichuan,

PR China

^5^Department of Radiology, Taihe Hospital, Hubei University of Medicine, Shiyan 442012, PR China

^6^Department of Radiology, West China Xiamen Hospital of Sichuan University, Xiamen 361021, Fujian, PR China.

**
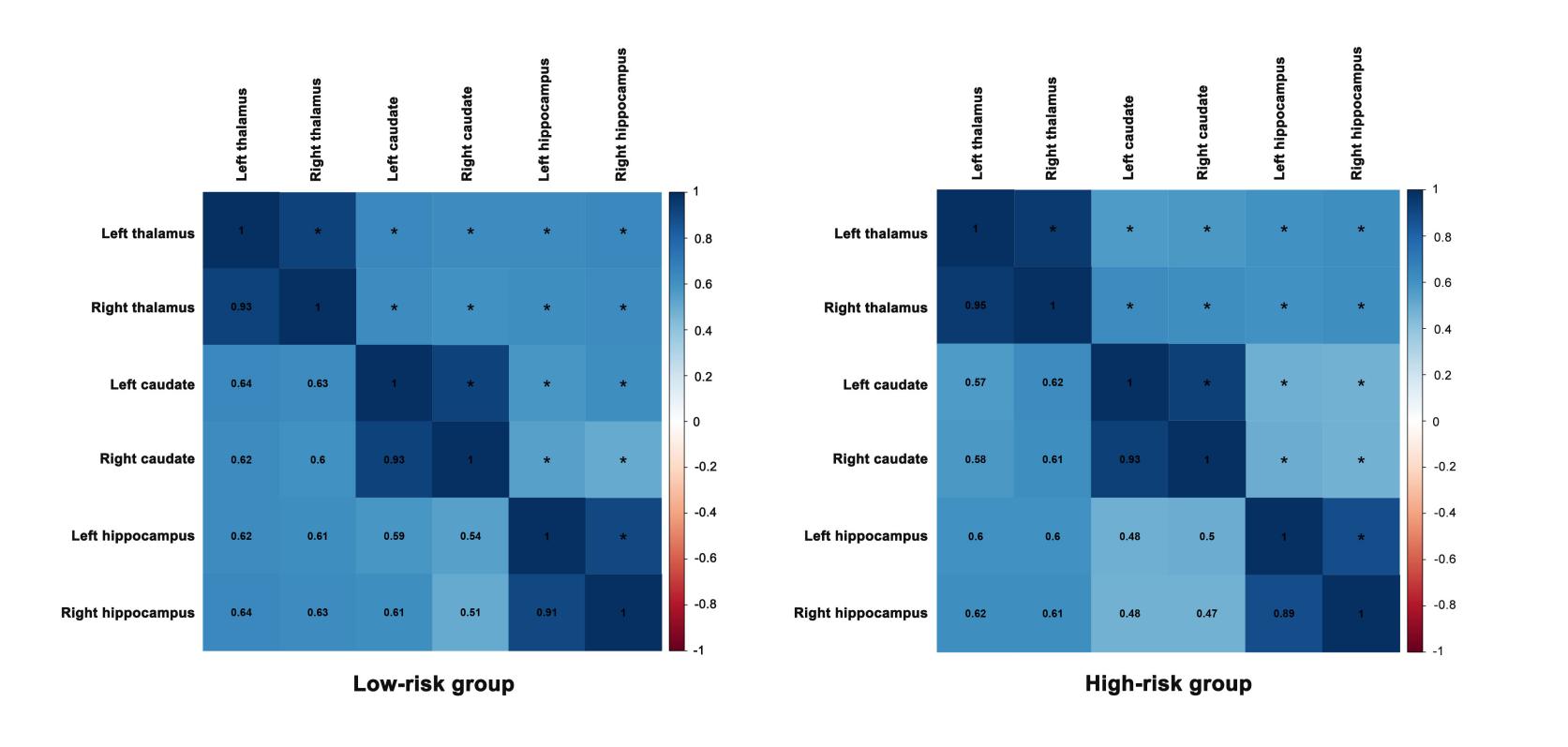
**

**Figure S1. The correlation matrices between the brain regions with significant differences in subcortical volumes across subjects within each group.** The matrix shows the Pearson correlation coefficients between the volumes of the brain regions with significant differences between the Low-risk and High-risk groups. The matrix is color-coded according to the strength and direction of the correlation, with blue indicating positive correlation and red indicating negative correlation. The darker the color, the stronger the correlation. The number in the bottom left corner of the correlation matrix represents the correlation coefficient between the measured values of the horizontal and vertical coordinates. The matrix also shows the p-values for each correlation coefficient, with asterisks indicating significance levels (*p<0.05, FDR-corrected).

**
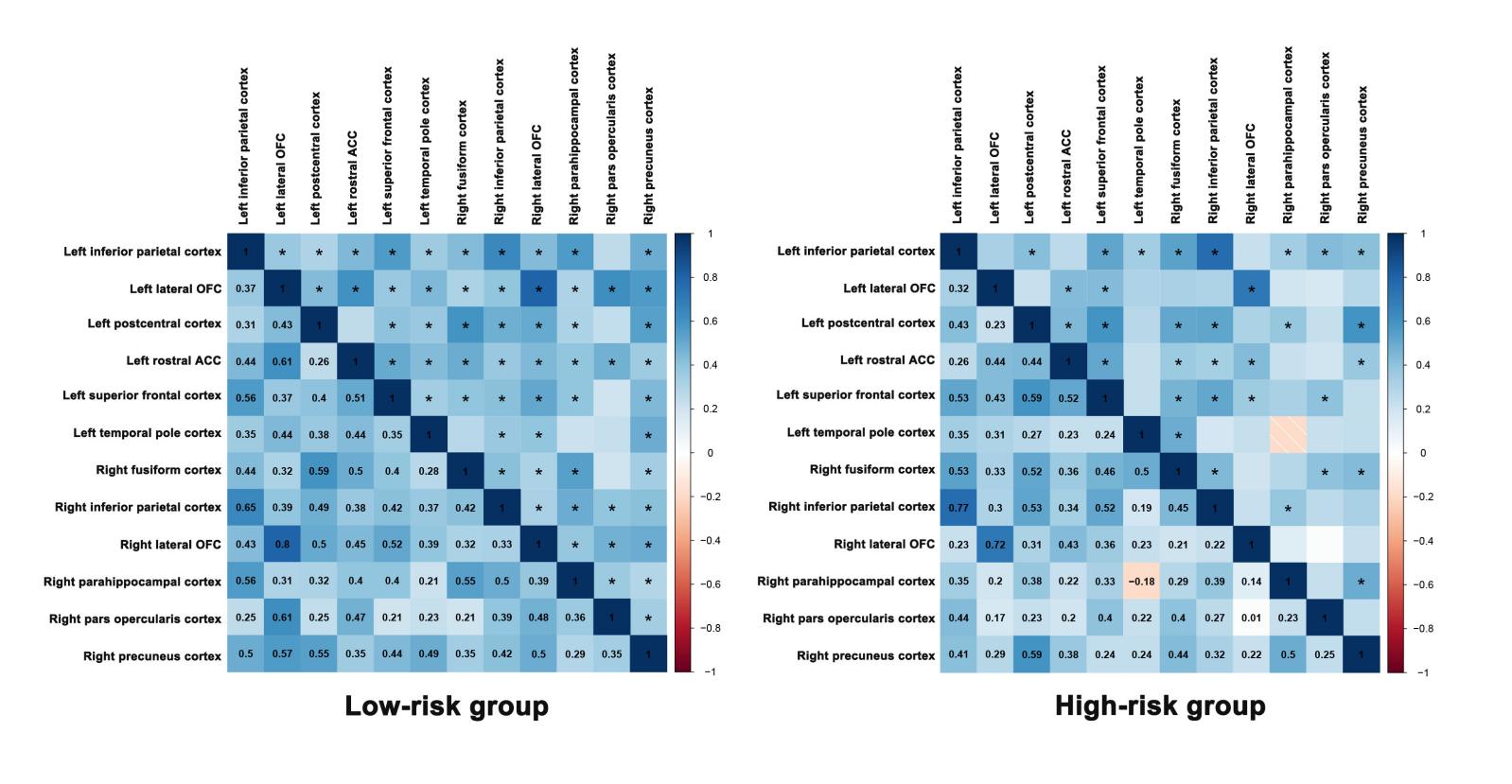
**

**Figure S2. The correlation matrices between the brain regions with significant differences in surface area across subjects within each group.** The matrix shows the Pearson correlation coefficients between the surface area of the brain regions with significant differences between the Low-risk and High-risk groups. The matrix is color-coded according to the strength and direction of the correlation, with blue indicating positive correlation and red indicating negative correlation. The darker the color, the stronger the correlation. The number in the bottom left corner of the correlation matrix represents the correlation coefficient between the measured values of the horizontal and vertical coordinates. The matrix also shows the p-values for each correlation coefficient, with asterisks indicating significance levels (*p<0.05, FDR-corrected). Abbreviations: OFC, orbitofrontal cortex; ACC, anterior cingulate cortex.
